# Serial quantification of CRP and total leukocyte count as a complementary tool in neonatal sepsis

**DOI:** 10.6026/97320630018920

**Published:** 2022-10-31

**Authors:** V Gomathi, G Chitrasivasankari, T Uma, MP Saravanan, Kaarthikeyan Gurumoorthy

**Affiliations:** 1 Institute of Biochemistry, Madras Medical College, RGGGH, Chennai, India; 2 Institute of Biochemistry, Stanley Medical College, Chennai, India; 3 Saveetha Dental College & Hospital, Saveetha Institute of Medical and Technical Sciences (SIMATS), Chennai - 77, India

**Keywords:** Neonatal sepsis, C-reactive protein, total leukocyte Count

## Abstract

Early detection and appropriate treatment of newborn sepsis reduce mortality and morbidity. A rapid, inexpensive laboratory approach is needed to assess newborn sepsis, even though blood culture is the gold standard for diagnosis. To compare serial CRP and Total Leukocyte Count (WBC) with blood culture, this study aimed to evaluate the role of newborn sepsis. A total 148 neonates with clinical symptoms of sepsis were included .CRP was measured by quantitative immuno turbidimetric method andotal leukocyte count (WBC) was measured by automated cell counter. CRP1 and WBC1 were measured within 6 hours of clinical symptoms. CRP2 and WBC2 were measured after 48 hours of clinical symptoms. Sensitivity, specificity, PPV, NPV of CRP1 and CRP2,WBC 1and WBC 2 were compared with culture positive and negative sepsis.CRP 2 showed high sensitivity 96% and high NPV95% with significant p value <0.0001. WBC2 has high sensitivity (90.57%) and NPV (91%) with significant p value <0.0001. CRP 1 has sensitivity 83%and NPV 82.3%, with p value < 0.001.WBC1 has lowest sensitivity (62.2%) and NPV (71.4%) compared to all other parameters. Serial CRP and WBC measurements are useful in the diagnosis of neonatal sepsis. Measurement of CRP and Total Leukocyte Count (WBC) after 48 hours of clinical symptoms were considered promptly for diagnose neonatal sepsis.

## Background:

Within the first four weeks of life, newborns can develop neonatal sepsis, a systemic infection [1]. The leading cause of neonatal mortality and morbidity in developing nations is neonatal sepsis, a dangerous illness that poses a serious risk to life. Neonatal sepsis can affect 1-24.5/1000 live births and 1-8 instances of all live births in India [2]. Neonatal sepsis is likely to cause 30 to 50 percent of neonatal deaths annually in underdeveloped nations [3-4]. Therefore, early identification and treatment are crucial for a successful outcome. The gold standard diagnosis is blood culture. However, it is not available in all peripheral centers, it is expensive and the findings are not always available right once. Numerous infants with sepsis-related clinical signs and symptoms have blood cultures that are frequently negative. Hence quick convenient, affordable, laboratory methods are required to evaluate neonatal sepsis. Many investigators have evaluated new markers like pro-calcitonin, cytokines, cell surface antigens for rapid diagnosis of sepsis, but their use in routine practice are limited by the lack of resources in developing countries [5-7] .C -Reactive protein and Total leukocyte counts (WBC) are frequently used for diagnosing neonatal sepsis, but a single value of CRP and WBC alone not sufficient to include and exclude sepsis. Therefore, it is of interest to assess the role of serial CRP and Total Leukocyte Count (WBC) in neonatal sepsis and to compare with blood culture.

## Materials and Methods:

### Study population:

This cross-sectional study was carried out at the Government Stanley Medical College and Hospital, Neonatal Intensive Care Unit (NICU), Department of Pediatrics, Department of Biochemistry and RSRM. The institutional ethical committee's clearance was obtained. The study involved 148 newborns admitted to the NICU with sepsis-related clinical signs. The neonates were included in the study after parents or guardians gave informed consent based on the inclusion and exclusion criteria listed below. All newborns admitted to the NICU with a clinical suspicion of neonatal sepsis in accordance with WHO integrated criteria were included [8]. Infants receiving antibiotics before examination and patients who have already had outside treatment have been sent to our facility. Blood samples were taken under rigorous aseptic precaution following the NICU's standard operating procedure for the following laboratory parameters. Following that, tests were conducted.

### CRP 1 and CRP 2:

CRP was divided into CRP1 and CRP2 categories based on the time of sample collection. CRP1 time of sample collection was completed within six hours of the onset of clinical sepsis signs. CRP2 time of sample collection was completed following 48 hours of sepsis-related clinical symptoms.

### Total leukocyte Count1 (WBC1) andTotal leucocyte Count2 (WBC2):

WBC1- Time of sample collection within 6 hours of the onset of sepsis-related clinical signs. WBC2 time of sample collection was completed following 48 hours of sepsis-related clinical symptoms.

### Blood culture:

#### Estimation of CRP:

In a red top tube, 2 cc of blood were drawn for the CRP measurement. The serum was separated and centrifuged at 2000-3000 rpm for 15 minutes after it had been allowed to clot for 20 minutes. Following that, serum was extracted and its amount calculated using an automated analyzer and an immuno-turbidimetric technique CRP, >5 mg/L, is regarded as positive (normal range, 2-5 mg/L).

#### Total leukocyte count:

1ml of blood was taken in EDTA tube and estimated by five part Automated Sysmex haematology analyzer. Total leucocyte Count (WBC), <5000/µL or >15000/µL considered as positive (Normal range 5000-15000µL). When there was a clinical suspicion of sepsis, 1 ml of blood was taken in a culture vial under aseptic conditions. Brain Heart Infusion broth (BHI) is contained in blood culture bottles in a 1:10 blood to BHI ratio. On day 1, day 3, and day seven, subsequent sub-cultures were performed on 5 percent sheep blood agar, chocolate agar, and Maccon key agar. Following the Clinical Laboratory Standard Institute (CLSI) recommendations, microorganisms were identified. Blood cultures were considered positive when the same microbe with the same antimicrobial sensitivity developed in both samples within 72 hours after collection.

The National Neonatology Forum's definition of sepsis for hospitals is based on culture [9]. Newborns confirmed sepsis (Culture positive) or culture verified sepsis is the first sepsis category. The second group included the Possibility of sepsis or clinical sepsis (Culture negative). In light of the results of the blood culture, two groups of newborns were created.

[1] Culture-proven sepsis is defined as sepsis with culture-positive results and sepsis-related clinical signs.

[2] Sepsis with culture-negative results and clinical signs of the disease (probable sepsis)

Sensitivity, specificity, PPV, NPV of CRP 1&2 and WBC 1&2 compared with gold standard test (blood culture)

## Results:

This study examined 148 newborns who had sepsis-related clinical signs. In all infants exhibiting clinical signs of sepsis, CRP 1, CRP 2, Total Leukocyte count 1 (WBC1), Total Leukocyte count 2 (WBC2), and blood culture were conducted. The total leukocyte count, WBC (1&2), blood culture, and CRP (1&2) findings were entered into excel sheets, and SPSS 16 software was used to conduct the statistical analysis. The Chi-square test is one of the statistical tests used for comparison. A p-value of 0.05 was considered significant. In addition to CRP1, CRP2, and WBC1 & WBC2, sensitivity, specificity, PPV, and NPV calculations were made.

## CRP 1 & blood culture:

Out of 148 neonates with clinical symptoms of sepsis, 97 (65.54%) neonates had CRP 1 that was positive (>5mg/L), while 51 (34.45%) of those same neonates had negative CRP (5mg/L). Fifty-three positive blood cultures were found. Out of 95 infants with negative blood cultures, 44 (83%) had positive CRP1, and 53 (55.7%) had positive CRP1. As a result, the correlation between CRP1 and the blood culture results was statistically significant (p-value 0.001).

## CRP 2 & blood culture:

Out of 148 newborns with clinical signs of sepsis, 108 (72.9%) had positive CRP 2 results (>5mg/L), while 40 (27.0%) had negative CRP 2 results (5mg/L). One hundred forty-eight neonates had sepsis-related clinical signs. CRP 2 was positive in 51 (96.2%) of the 53 infants with positive blood cultures and 57 (60%) of the 95 neonates with negative blood cultures. As a result, the relationship between CRP2 and the blood culture results was statistically significant (p-value 0.0001).

## Total leukocyte count (WBC) 1 & 2 with blood culture:

Out of 148 infants with clinical signs of sepsis, total leukocyte count 1 (WBC1) was positive (5000/L or >15000 L) in 78 (52.7%) neonates and normal (5000-15000 L) in 70 (47.3%) neonates. WBC1 was positive in 33 (62%) of the 53 neonates with sepsis with positive blood cultures. WBC was positive in 45 (47%) of the 95 newborns with sepsis who had negative blood cultures. With a p-value of 0.1, the test result was not statistically significant. Out of 148 neonates with clinical signs of sepsis, 89 (or 60%) had WBC 2 that was positive (5000–15000 L), and 59 (or 40%) had it normal? WBC2 was positive in 48 (90 percent) of the 53 newborns with positive blood cultures for sepsis. Out of 95 neonates with negative blood cultures, 41 (43%) had positive WBC2 results. With a p-value of 0.0001, this test result was statistically significant.

Table 1(see PDF) displays the baseline characteristics of the study population. It shows 148 neonates with clinical signs & symptoms of sepsis were included in this study, out of 148 neonates with clinical signs & symptoms of sepsis 90(60.8%) were male and 58 (39.2%) were female. 85 (57.44%)were delivered through assisted deliveries, and 63(42.5%) were delivered through vaginal route. Based on maturity 81(54.7%) were pre term babies, 67 (45.2%) were term babies. Regarding birth weight 76(51.3%) were Low birth weight (LBW), 72 (48.6%) were normal weight.53 (35.8%) were blood culture positive, 95 (64.1%) was negative on blood culture.Table 2(see PDF) explains percentage of male neonates preterm babies LBW babies were high in culture proven sepsis compared with culture negative sepsis which was statistically significant p vale <0.05. Table 3(see PDF) describes percentage of microorganism found in blood culture Acinetobacter was the most common organism isolated followed by Klebsiella and Coagulus negative Staphylococcus aureus, Staphlococcus aureus and Enterobacter, Pseudomonas, E. coli and Citrobacter were less commonly present. Table 4(see PDF) shows comparison of CRP1,CRP2 ,TOTAL LEUCOCYTE COUNT (WBC) 1&2 with Gold standard blood culture. Table 5(see PDF) shows CRP2 has highest sensitivity 96% and NPV 95%, next toCRP2, Total Leukocyte Count 2(wbc2) has high sensitivity 90.57%, NPV 91% .CRP1 has83% sensitivity and NPV 82.3%. WBC1 has lowest sensitivity 62.2%, NPV 71.4%.

## Discussion:

The current study included 148 neonates brought to the neonatal critical care unit with clinical signs of sepsis. Ninety newborns (60.8 percent) were male, and 58 (39.2 percent) were female of the 148 neonates. Ninety-five neonates (65.18%) and 53 (35.18%) neonates, respectively, have sepsis that has been confirmed by culture. Fifty-three neonates with positive blood cultures were divided into 45 (84 percent) males and 8 (16 percent) females. It might be because males have an X-linked immune-regulatory gene component that makes them more susceptible to infections [10]. 33 (67%) of the study's LBW newborns were positive for culture. This is brought on by the infection rate, negatively correlated with newborns' birth weights, low levels of immunoglobulin G and weakened cellular immunity. Similar findings were discovered in research by Barbara Stoll et al. [11]. 66 percent of preterm infants have sepsis that has been verified in a culture. This is higher than the sepsis rate in culture-proven term newborns (44 percent). This is due to cellular and humoral immunity's inherent limitations. Incidence of septicemia is inversely correlated with neonatal gestational age. According to Barbara J. Stoll et al. study, Acinetobacter predominated in the blood culture organisms, followed by Klebsiella. One of the newly emerging possible pathogens in neonatal septicemia that has been commonly discovered in recent years is Acinetobacter (gram-negative bacteria) [12-14].In comparison to culture results, CRP 2 has a significant p-value of 0.0001 and high sensitivity of 96%, and NPV of 95%. This research is most in line with Pal et al. Jadhav et al. and Chacha et al. [15-17]. CRP 1 has an NPV of 82.3 percent, a sensitivity of 83 percent, and a p-value of 0.001. Based on sensitivity and strong negative predictive value in neonatal sepsis, measurement of CRP after 48 hours reveals higher sensitivity and NPV than earlier measurement, demonstrating that CRP 2 is a good diagnostic tool for ruling in sepsis and ruling out sepsis [17- 18]. Within six hours of the start of an infectious process, the liver begins to manufacture C-reactive protein, an acute-phase reactant protein [19-20]. A single CRP measurement within 6 hours of clinical suspicion of sepsis is insufficient to diagnose neonatal septicemia. This is because, unlike physiological conditions like intra ventricular hemorrhage, stressful deliveries, fetal distress, meconium aspiration, and perinatal asphyxia, the CRP value increases and returns to normal in 24 to 48 hours. The CRP value remains elevated in an infectious condition even after 48 hours. Therefore, measuring CRP after 48 hours is necessary to improve sensitivity and eliminate false positive results. In this study, CRP was assessed twice: once within six hours of the onset of clinical sepsis symptoms and again 48 hours later. It was discovered that CRP 2 is a more sensitive indication than CRP 1. These findings are comparable to those of other studies [17, 18, 21]. Total Leukocyte Count 1 has a low sensitivity (62.2 percent) and NPV (71.4 percent) when performed within 6 hours of clinical symptoms. Because these values are initially normal in the early period, it has been determined that other studies show that Total Leukocyte count (WBC) has little correlation with neonatal septicemia and is not a reliable indicator of infection during the first few hours of infection [21, 22, 24]. Total Leucocyte Count 2, on the other hand, exhibits great sensitivity (90.57 percent) and NPV (91 percent) after 48 hours of clinical sepsis symptoms. These findings are most in line with earlier research [25].

## Conclusions:

CRP and Total Leukocyte measurement after 48 hours of clinical symptoms of sepsis enhance the sensitivity and NPV in neonatal sepsis compared with initial assay (with in 6 hrs). Serial measurement of CRP and Total leukocyte count (WBC) neonates with clinical symptoms can be considered promptly for diagnosis of neonatal sepsis would be rather than single measurement.

## References

[1] Ansari S (2015). International Journal of Pediatrics.

[2] Panwar C (2017). Int J Contemp Pediatr.

[3] Vijayan S (2020). Int J Contemp Pediatr..

[4] Vergnano S (2005). Archives of Disease in Childhood-Fetal and Neonatal Edition..

[5] Buch AC (2011). International Journal of Basic and Applied Medical Sciences..

[6] Bhat Ry (2010). Journal of Clinical and Diagnostic Research..

[7] Gessner B (2005). Epidemiol Infect..

[8] Group WY (1999). Pediatr Infect Dis J.

[9] https://www.jaypeedigital.com/book/9788180614217.

[10] Stoll B (2010). Pediatrics..

[11] De AS (2013). J Glob Infect Dis..

[12] Bhat A (2016). International Journal of Medical and Dental Sciences.

[13] Zhou B, Liu X (2016). ExpTher Med..

[14] Jaswal RS (2003). Indian Pediatr..

[15] Bangi V, Devi S (2014). J Ntr Univ Health Sci..

[16] Chacha F (2014). BMC Pediatrics..

[17] Ghosh S (2001). Indian J Med Sci..

[18] Chiesa C (2004). Clin Chem.

[19] Hofer N (2012). Neonatology.

[20] Franz AR (1999). Pediatr Infect Dis J.

[21] Ohlsson A (1987). Am J ObstetGynecol.

[22] Yoon et (1996). Obstet Gynecol.

[23] Sundarapandian S (2017). Indian Journal of Neonatal Medicine and Research.

[24] Zhou B (2016). Exp Ther Med.

[25] Du J (2014). PLoS One.

